# (2-Decan­amido­eth­yl)dimethyl­amine *N*-oxide

**DOI:** 10.1107/S1600536810022270

**Published:** 2010-06-23

**Authors:** Agnieszka Lewińska

**Affiliations:** a Faculty of Chemistry, Wrocław University, ul. Joliot-Curie 14, 50-383 Wrocław, Poland

## Abstract

In the title compound, C_14_H_30_N_2_O_2_, the almost planar nonyl chains are fully extended: the N—C—C—N torsion angle of −161.95 (8)° indicates an *anti* conformation. The crystal structure features N—H⋯O hydrogen bonds and C—H⋯O inter­actions.

## Related literature

For the bond lengths and angles of nonyl chains, see: Low *et al.* (1999 ▶); Kato & Ikemori (2003 ▶); Ulrich *et al.* (1990 ▶). For related structures containing the amide group, see: Belicchi-Ferrari *et al.* (2007 ▶); Jeffrey & Maluszynska (1989 ▶). For N—O bond lengths, see: Katrusiak *et al.* (1987 ▶); Kemmitt *et al.* (2002 ▶); Maia *et al.* (1984 ▶); Boese *et al.* (1999 ▶); Palatinus & Damay (2009 ▶). For a related structure, see: Sauer *et al.* (2003 ▶). For the synthesis, see: Piłakowska-Pietras *et al.* (2008 ▶). For hydrogen-bond motifs, see: Bernstein *et al.* (1995 ▶); Rospenk *et al.* (1989 ▶). 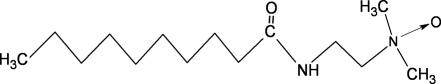


## Experimental

### 

#### Crystal data

C_14_H_30_N_2_O_2_
*M* *_r_* = 258.40
Triclinic, 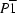

*a* = 5.378 (2) Å
*b* = 8.113 (4) Å
*c* = 17.801 (5) Å
α = 79.55 (4)°
β = 86.38 (3)°
γ = 86.36 (4)°
*V* = 761.2 (5) Å^3^
*Z* = 2
Mo *K*α radiation
μ = 0.08 mm^−1^
*T* = 100 K
0.23 × 0.19 × 0.08 mm

#### Data collection

Oxford Diffraction Xcalibur Sapphire2 diffractometer
10305 measured reflections
3149 independent reflections
2746 reflections with *I* > 2σ(*I*)
*R*_int_ = 0.018

#### Refinement

*R*[*F* ^2^ > 2σ(*F* ^2^)] = 0.033
*wR*(*F* ^2^) = 0.094
*S* = 1.06
3149 reflections
169 parameters
H atoms treated by a mixture of independent and constrained refinement
Δρ_max_ = 0.33 e Å^−3^
Δρ_min_ = −0.16 e Å^−3^

### 

Data collection: *CrysAlis CCD* (Oxford Diffraction, 2007 ▶); cell refinement: *CrysAlis RED* (Oxford Diffraction, 2007 ▶); data reduction: *CrysAlis RED*; program(s) used to solve structure: *SHELXTL* (Sheldrick, 2008 ▶); program(s) used to refine structure: *SHELXTL*; molecular graphics: *SHELXTL*; software used to prepare material for publication: *SHELXTL*.

## Supplementary Material

Crystal structure: contains datablocks I, global. DOI: 10.1107/S1600536810022270/ds2034sup1.cif

Structure factors: contains datablocks I. DOI: 10.1107/S1600536810022270/ds2034Isup2.hkl

Additional supplementary materials:  crystallographic information; 3D view; checkCIF report

## Figures and Tables

**Table 1:** Hydrogen-bond geometry (Å, °)

*D*—H⋯*A*	*D*—H	H⋯*A*	*D*⋯*A*	*D*—H⋯*A*
N2—H2⋯O1^i^	0.87 (2)	1.89 (2)	2.753 (2)	168.9 (2)
C1—H1*A*⋯O2^ii^	0.99	2.48	3.453 (2)	166
C1—H1*B*⋯O2^iii^	0.99	2.47	3.363 (2)	150
C4—H4*A*⋯O1^i^	0.99	2.32	3.204 (2)	148
C13—H13*C*⋯O2^iii^	0.98	2.58	3.438 (2)	146
C14—H14*B*⋯O2^iii^	0.98	2.60	3.449 (2)	145

Symmetry codes: (i) 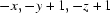; (ii) 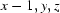; (iii) 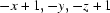.

**Fig. 1.**
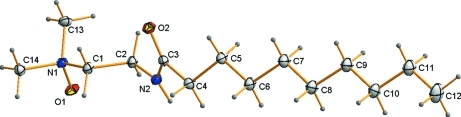
Table 1. Selected geometric parameters (Å, °).

**Fig. 2.**
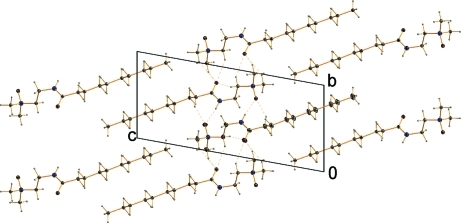
Table 2. Hydrogen bond parameters (Å, °).

## supplementary crystallographic information

### Comment

Surfactants are amphiphilic molecules composed by at least two parts, one of them is polar or hydrophilic and the other one nonpolar or hydrophobic. A special group of surface active amine oxides are amidoamine oxides based on fatty monocarboxylic acids and diamines, particularly *N*,*N*-dimethylethylenediamine and *N*,*N*-dimethyl-1,3-propanediamine. These surfactants are typically employed in hair and body care, cleaning and shampoo formulations as foaming agents, wetting agents, thickeners and conditioners They are low or nontoxic to humans and higherorganisms but at the same time exhibit an antimicrobial activity.

The crystal and molecular structure of typical N-oxide derivatives were previously determined for 17-oxosparteine N(*l*)-oxide hydrochloride (A. Katrusiak, *et al.*) and 4-methylpyridine-N-oxide (L.Palatinus *et al*.). The crystal and molecular structure recognized for N-oxide surfactant, *N*,*N*-dimethyl-n-tetradecylamine oxide (Fronczek *et al. *), in some degree is similar to the structure of our compound. In general, N-oxide derivatives and especially N-oxide surfactants are known as very difficult for crystallization, so the crystal structure solution for 2-(decanoylamino)ethyldimethylamine-N-oxide presented in this report is a very rare case.

The title compound consists of a hydrophobic alkyl chain and a lipophilic moiety represented by amide and N-oxide groups bridged by ethyl group (Figure 1). The planar nine carbon side adopt fully extended conformations and is twisted 45.6 (1)° from the plane of adjacent amide moiety. The torsion angle N1—C1—C2—N2 of -161.95 (8)° shows that this part takes an antiperiplanar conformation. The bond lengths and angles of nonyl chain Low *et al.* (1999) and amide group Belicchi-Ferrari *et al. * (2007) are within the normal ranges and comparable to the previously reported structures. The N—O bond length of is slightly shorter than the corresponding distances in tertiary acyclic amine oxides Boese *et al.* (1999).

The crystal structures is composed of the alternated hydrophilic and hydrophobic layers (Figure 1). The components in the hydrophilic parts are linked to each other *via* N—H···O bonds of R2,2(10) ring motifs Ulrich *et al. * (1990) and the weak C—H···O interactions (Table 2), whereas in the hydrophobic regions they interact through van der Waals contacts.

### Experimental

A title compound was synthesized according to method given by Piłakowska-Pietras *et al.* (2008) The surfactant was carefully purificated several times. Suitable single crystalwere obtained by slow evaporation of thecompoundsolutionin a chloroform–hexane mixture and kept cold at -5¯C. The crystals of 2-(decanoylamino)ethyldimethylamine-N-oxides appeared unexpectedly taking into account well known problems with the surfactants crystallization.

### Refinement

All the H atoms were positioned geometrically and refined using a riding model with C—H = 0.98–0.99 Å. The *U*_iso_ values were constrained to be -1.5U_equ_ (methyl H atoms) and -1.2U_equ_ (other H atoms). The rotating model group was considered for the methyl group. In the case of N1, the hydrogen atom was located from a difference Fourier map and refined isotropically.

### Crystal data

**Table:**

C_14_H_30_N_2_O_2_	*Z* = 2
*M**_r_* = 258.40	*F*(000) = 288
Triclinic, *P*1	*D*_x_ = 1.127 Mg m^−^^3^
Hall symbol: -P 1	Mo *K*α radiation, λ = 0.71073 Å
*a* = 5.378 (2) Å	Cell parameters from 9030 reflections
*b* = 8.113 (4) Å	θ = 3–36°
*c* = 17.801 (5) Å	µ = 0.08 mm^−^^1^
α = 79.55 (4)°	*T* = 100 K
β = 86.38 (3)°	Block, colorless
γ = 86.36 (4)°	0.23 × 0.19 × 0.08 mm
*V* = 761.2 (5) Å^3^	

### Data collection

**Table:**

Oxford Diffraction Xcalibur Sapphire2 (large Be window) diffractometer	2746 reflections with *I* > 2σ(*I*)
Radiation source: fine-focus sealed tube	*R*_int_ = 0.018
graphite	θ_max_ = 26.5°, θ_min_ = 3.0°
ω scans	*h* = −6→6
10305 measured reflections	*k* = −8→10
3149 independent reflections	*l* = −22→22

### Refinement

**Table:**

Refinement on *F*^2^	Primary atom site location: structure-invariant direct methods
Least-squares matrix: full	Secondary atom site location: difference Fourier map
*R*[*F*^2^ > 2σ(*F*^2^)] = 0.033	Hydrogen site location: inferred from neighbouring sites
*wR*(*F*^2^) = 0.094	H atoms treated by a mixture of independent and constrained refinement
*S* = 1.06	*w* = 1/[σ^2^(*F*_o_^2^) + (0.0535*P*)^2^ + 0.1328*P*] where *P* = (*F*_o_^2^ + 2*F*_c_^2^)/3
3149 reflections	(Δ/σ)_max_ = 0.001
169 parameters	Δρ_max_ = 0.33 e Å^−^^3^
0 restraints	Δρ_min_ = −0.15 e Å^−^^3^

### Special details

**Table:**

Geometry. All e.s.d.'s (except the e.s.d. in the dihedral angle between two l.s. planes) are estimated using the full covariance matrix. The cell e.s.d.'s are taken into account individually in the estimation of e.s.d.'s in distances, angles and torsion angles; correlations between e.s.d.'s in cell parameters are only used when they are defined by crystal symmetry. An approximate (isotropic) treatment of cell e.s.d.'s is used for estimating e.s.d.'s involving l.s. planes.
Refinement. Refinement of *F*^2^ against ALL reflections. The weighted *R*-factor *wR* and goodness of fit *S* are based on *F*^2^, conventional *R*-factors *R* are based on *F*, with *F* set to zero for negative *F*^2^. The threshold expression of *F*^2^ > σ(*F*^2^) is used only for calculating *R*-factors(gt) *etc*. and is not relevant to the choice of reflections for refinement. *R*-factors based on *F*^2^ are statistically about twice as large as those based on *F*, and *R*- factors based on ALL data will be even larger.

### Fractional atomic coordinates and isotropic or equivalent isotropic displacement parameters (Å^2^)

**Table:**

	*x*	*y*	*z*	*U*_iso_*/*U*_eq_	
O1	−0.14655 (12)	0.29531 (8)	0.63607 (4)	0.01841 (17)	
O2	0.61547 (12)	0.19768 (8)	0.42458 (4)	0.01908 (17)	
N1	−0.00050 (14)	0.15787 (9)	0.61997 (4)	0.01399 (18)	
N2	0.31670 (14)	0.39834 (10)	0.44253 (4)	0.01548 (18)	
C1	0.11161 (17)	0.19110 (11)	0.53929 (5)	0.0149 (2)	
H1A	−0.0245	0.2133	0.5033	0.018*	
H1B	0.2102	0.0895	0.5291	0.018*	
C2	0.27914 (17)	0.33925 (11)	0.52434 (5)	0.01533 (19)	
H2A	0.2015	0.4313	0.5490	0.018*	
H2B	0.4422	0.3047	0.5468	0.018*	
C3	0.48478 (16)	0.32380 (11)	0.39906 (5)	0.01460 (19)	
C4	0.49927 (17)	0.40400 (12)	0.31534 (5)	0.0169 (2)	
H4A	0.4090	0.5154	0.3094	0.020*	
H4B	0.4129	0.3340	0.2861	0.020*	
C5	0.76504 (17)	0.42604 (12)	0.28085 (5)	0.0175 (2)	
H5A	0.8587	0.4857	0.3128	0.021*	
H5B	0.8504	0.3144	0.2805	0.021*	
C6	0.76453 (18)	0.52507 (12)	0.19949 (5)	0.0187 (2)	
H6A	0.6695	0.6335	0.2000	0.022*	
H6B	0.6765	0.4620	0.1675	0.022*	
C7	1.02383 (18)	0.55974 (13)	0.16275 (6)	0.0210 (2)	
H7A	1.1189	0.4515	0.1617	0.025*	
H7B	1.1125	0.6227	0.1946	0.025*	
C8	1.01841 (18)	0.65973 (13)	0.08150 (5)	0.0217 (2)	
H8A	0.9321	0.5957	0.0496	0.026*	
H8B	0.9202	0.7668	0.0825	0.026*	
C9	1.27600 (19)	0.69820 (13)	0.04450 (6)	0.0218 (2)	
H9A	1.3607	0.7643	0.0759	0.026*	
H9B	1.3753	0.5911	0.0446	0.026*	
C10	1.27329 (19)	0.79467 (13)	−0.03721 (6)	0.0218 (2)	
H10A	1.1736	0.9017	−0.0377	0.026*	
H10B	1.1907	0.7284	−0.0690	0.026*	
C11	1.5337 (2)	0.83259 (13)	−0.07251 (6)	0.0246 (2)	
H11A	1.6154	0.8998	−0.0409	0.030*	
H11B	1.6338	0.7256	−0.0714	0.030*	
C12	1.5335 (2)	0.92734 (15)	−0.15446 (6)	0.0327 (3)	
H12A	1.7055	0.9478	−0.1738	0.049*	
H12B	1.4383	1.0348	−0.1559	0.049*	
H12C	1.4566	0.8605	−0.1865	0.049*	
C13	0.19656 (17)	0.11555 (12)	0.67652 (5)	0.0181 (2)	
H13A	0.1186	0.0964	0.7284	0.027*	
H13B	0.3077	0.2087	0.6709	0.027*	
H13C	0.2931	0.0138	0.6675	0.027*	
C14	−0.15989 (17)	0.01185 (11)	0.62702 (5)	0.0180 (2)	
H14A	−0.2401	−0.0103	0.6786	0.027*	
H14B	−0.0565	−0.0872	0.6179	0.027*	
H14C	−0.2881	0.0369	0.5892	0.027*	
H2	0.246 (2)	0.4950 (17)	0.4221 (7)	0.027 (3)*	

### Atomic displacement parameters (Å^2^)

**Table:**

	*U*^11^	*U*^22^	*U*^33^	*U*^12^	*U*^13^	*U*^23^
O1	0.0201 (3)	0.0152 (3)	0.0181 (3)	0.0071 (3)	0.0033 (3)	−0.0022 (3)
O2	0.0176 (3)	0.0169 (3)	0.0207 (4)	0.0037 (3)	0.0009 (3)	−0.0003 (3)
N1	0.0136 (4)	0.0137 (4)	0.0135 (4)	0.0021 (3)	0.0010 (3)	−0.0008 (3)
N2	0.0151 (4)	0.0140 (4)	0.0154 (4)	0.0010 (3)	0.0015 (3)	0.0013 (3)
C1	0.0156 (4)	0.0163 (4)	0.0119 (4)	−0.0004 (3)	0.0012 (3)	−0.0013 (3)
C2	0.0149 (4)	0.0163 (4)	0.0139 (4)	−0.0006 (3)	0.0010 (3)	−0.0007 (3)
C3	0.0124 (4)	0.0136 (4)	0.0174 (4)	−0.0024 (3)	0.0002 (3)	−0.0017 (3)
C4	0.0153 (4)	0.0182 (5)	0.0160 (5)	0.0009 (3)	0.0010 (3)	−0.0015 (3)
C5	0.0158 (4)	0.0186 (5)	0.0163 (5)	0.0011 (3)	0.0026 (3)	−0.0002 (4)
C6	0.0180 (5)	0.0206 (5)	0.0159 (5)	−0.0002 (4)	0.0019 (4)	−0.0005 (4)
C7	0.0189 (5)	0.0248 (5)	0.0170 (5)	−0.0003 (4)	0.0024 (4)	0.0012 (4)
C8	0.0209 (5)	0.0260 (5)	0.0161 (5)	−0.0018 (4)	0.0018 (4)	0.0013 (4)
C9	0.0217 (5)	0.0244 (5)	0.0171 (5)	−0.0020 (4)	0.0021 (4)	0.0012 (4)
C10	0.0239 (5)	0.0236 (5)	0.0165 (5)	−0.0029 (4)	0.0015 (4)	0.0001 (4)
C11	0.0267 (5)	0.0266 (5)	0.0183 (5)	−0.0032 (4)	0.0038 (4)	0.0007 (4)
C12	0.0408 (7)	0.0354 (6)	0.0193 (5)	−0.0086 (5)	0.0057 (5)	0.0016 (4)
C13	0.0177 (4)	0.0208 (5)	0.0145 (4)	0.0018 (4)	−0.0030 (3)	0.0001 (4)
C14	0.0157 (4)	0.0168 (5)	0.0201 (5)	−0.0021 (3)	0.0027 (4)	−0.0008 (4)

### Geometric parameters (Å, °)

**Table:**

O1—N1	1.385 (2)	C5—H5A	0.9900
O2—C3	1.237 (2)	C5—H5B	0.9900
N1—C1	1.506 (2)	C6—H6A	0.9900
N1—C13	1.489 (2)	C6—H6B	0.9900
N1—C14	1.489 (2)	C7—H7A	0.9900
N2—C2	1.454 (2)	C7—H7B	0.9900
N2—C3	1.340 (2)	C8—H8A	0.9900
N2—H2	0.87 (2)	C8—H8B	0.9900
C1—C2	1.523 (2)	C9—H9A	0.9900
C3—C4	1.514 (2)	C9—H9B	0.9900
C4—C5	1.528 (2)	C10—H10A	0.9900
C5—C6	1.523 (2)	C10—H10B	0.9900
C6—C7	1.524 (2)	C11—H11A	0.9900
C7—C8	1.525 (2)	C11—H11B	0.9900
C8—C9	1.522 (2)	C12—H12A	0.9800
C9—C10	1.522 (2)	C12—H12B	0.9800
C10—C11	1.524 (2)	C12—H12C	0.9800
C11—C12	1.520 (2)	C13—H13A	0.9800
C1—H1A	0.9900	C13—H13B	0.9800
C1—H1B	0.9900	C13—H13C	0.9800
C2—H2A	0.9900	C14—H14A	0.9800
C2—H2B	0.9900	C14—H14B	0.9800
C4—H4A	0.9900	C14—H14C	0.9800
C4—H4B	0.9900		
			
O1—N1—C1	111.04 (7)	C7—C6—H6B	109.00
O1—N1—C13	109.25 (7)	H6A—C6—H6B	108.00
O1—N1—C14	108.99 (7)	C6—C7—H7A	109.00
C1—N1—C13	111.29 (7)	C6—C7—H7B	109.00
C1—N1—C14	107.63 (7)	C8—C7—H7A	109.00
C13—N1—C14	108.58 (8)	C8—C7—H7B	109.00
C2—N2—C3	122.22 (8)	H7A—C7—H7B	108.00
C3—N2—H2	117.9 (8)	C7—C8—H8A	109.00
C2—N2—H2	119.1 (8)	C7—C8—H8B	109.00
N1—C1—C2	112.89 (8)	C9—C8—H8A	109.00
N2—C2—C1	110.07 (8)	C9—C8—H8B	109.00
O2—C3—N2	123.01 (9)	H8A—C8—H8B	108.00
O2—C3—C4	122.25 (9)	C8—C9—H9A	109.00
N2—C3—C4	114.73 (8)	C8—C9—H9B	109.00
C3—C4—C5	114.08 (8)	C10—C9—H9A	109.00
C4—C5—C6	110.99 (8)	C10—C9—H9B	109.00
C5—C6—C7	113.98 (8)	H9A—C9—H9B	108.00
C6—C7—C8	112.97 (8)	C9—C10—H10A	109.00
C7—C8—C9	113.64 (8)	C9—C10—H10B	109.00
C8—C9—C10	114.14 (9)	C11—C10—H10A	109.00
C9—C10—C11	112.91 (8)	C11—C10—H10B	109.00
C10—C11—C12	113.41 (9)	H10A—C10—H10B	108.00
N1—C1—H1A	109.00	C10—C11—H11A	109.00
N1—C1—H1B	109.00	C10—C11—H11B	109.00
C2—C1—H1A	109.00	C12—C11—H11A	109.00
C2—C1—H1B	109.00	C12—C11—H11B	109.00
H1A—C1—H1B	108.00	H11A—C11—H11B	108.00
N2—C2—H2A	110.00	C11—C12—H12A	109.00
N2—C2—H2B	110.00	C11—C12—H12B	109.00
C1—C2—H2A	110.00	C11—C12—H12C	109.00
C1—C2—H2B	110.00	H12A—C12—H12B	110.00
H2A—C2—H2B	108.00	H12A—C12—H12C	109.00
C3—C4—H4A	109.00	H12B—C12—H12C	109.00
C3—C4—H4B	109.00	N1—C13—H13A	109.00
C5—C4—H4A	109.00	N1—C13—H13B	109.00
C5—C4—H4B	109.00	N1—C13—H13C	110.00
H4A—C4—H4B	108.00	H13A—C13—H13B	109.00
C4—C5—H5A	109.00	H13A—C13—H13C	109.00
C4—C5—H5B	109.00	H13B—C13—H13C	109.00
C6—C5—H5A	109.00	N1—C14—H14A	109.00
C6—C5—H5B	109.00	N1—C14—H14B	109.00
H5A—C5—H5B	108.00	N1—C14—H14C	109.00
C5—C6—H6A	109.00	H14A—C14—H14B	110.00
C5—C6—H6B	109.00	H14A—C14—H14C	109.00
C7—C6—H6A	109.00	H14B—C14—H14C	109.00
			
O1—N1—C1—C2	60.52 (9)	N2—C3—C4—C5	135.19 (9)
C13—N1—C1—C2	−61.41 (9)	C3—C4—C5—C6	−173.49 (8)
C14—N1—C1—C2	179.74 (8)	C4—C5—C6—C7	177.04 (8)
C3—N2—C2—C1	−81.38 (10)	C5—C6—C7—C8	−179.74 (9)
C2—N2—C3—O2	1.92 (13)	C6—C7—C8—C9	178.94 (9)
C2—N2—C3—C4	−179.36 (8)	C7—C8—C9—C10	178.76 (9)
N1—C1—C2—N2	−161.95 (8)	C8—C9—C10—C11	179.52 (9)
O2—C3—C4—C5	−46.08 (12)	C9—C10—C11—C12	179.38 (9)

### Hydrogen-bond geometry (Å, °)

**Table:**

*D*—H···*A*	*D*—H	H···*A*	*D*···*A*	*D*—H···*A*
N2—H2···O1^i^	0.87 (2)	1.89 (2)	2.753 (2)	168.9 (2)
C1—H1A···O2^ii^	0.99	2.48	3.453 (2)	166
C1—H1B···O2^iii^	0.99	2.47	3.363 (2)	150
C4—H4A···O1^i^	0.99	2.32	3.204 (2)	148
C13—H13C···O2^iii^	0.98	2.58	3.438 (2)	146
C14—H14B···O2^iii^	0.98	2.60	3.449 (2)	145

Symmetry codes: (i) −*x*, −*y*+1, −*z*+1; (ii) *x*−1, *y*, *z*; (iii) −*x*+1, −*y*, −*z*+1.
